# B-glucans from Grifola frondosa and Ganoderma lucidum in breast cancer: an example of complementary and integrative medicine

**DOI:** 10.18632/oncotarget.24984

**Published:** 2018-05-15

**Authors:** Paola Rossi, Raffaele Difrancia, Vincenzo Quagliariello, Elena Savino, Paolo Tralongo, Cinzia Lucia Randazzo, Massimiliano Berretta

**Affiliations:** ^1^ Department of Biology and Biotechnology “L. Spallanzani”, University of Pavia, Italy; ^2^ Gruppo Oncologico Ricercatori Italiani, GORI onlus, Pordenone, Italy; ^3^ Department of Abdominal Oncology, National Cancer Institute, IRCCS - Foundation G. Pascale, Naples, Italy; ^4^ Department of Earth and Environmental Science, University of Pavia, Italy; ^5^ Oncology Division Umberto I Hospital, Siracusa, Italy; ^6^ Department of Agricultural, Food and Environment, University of Catania, Italy; ^7^ Department of Medical Oncology, National Cancer Institute, IRCCS, Aviano (PN), Italy

**Keywords:** G. lucidum, G. frondosa, breast cancer, immunomodulation, microbiota

## Abstract

Culinary and medicinal mushrooms are widely used in Asian countries, both as dietary supplements and as nutraceutical foods. They have recently become popular in Europe, as well, for their nutritional and health benefits. In particular, epidemiological studies conducted in Asia suggest that mushroom intake, together with other phytotherapy substances, protects against cancer, specifically gastrointestinal (GI) and breast cancers. Most of the data come from *in vitro* studies and *in vivo* experimental animal models. Therefore, in order to translate the updated knowledge to clinical research (i.e., from bench to bedside) a systematic translational research program should be initiated. Future randomized controlled trials comparing the effects of *G. frondosa* and *G. lucidum* on conventional treatment outcomes are warranted.

The purpose of this review was to describe the emerging mechanisms of action of the mushrooms’ anticancer functions which makes their use in clinical practice so promising. Clinical effects of mycotherapy (specifically, the use of *Ganoderma lucidum* and *Grifola frondosa*) on long-term survival, tumor response, host immune functions, inflammation, and QoL in cancer patients were also addressed. Adverse events associated with mycotherapy were also investigated. Emerging data point to a potential role of *G. lucidum* for modulating the carcinogenic potential of GI microbiota, which suggests a new complementary and integrated approach to breast cancer treatment.

## INTRODUCTION

Complementary and integrative medicine (CIM) is a relatively new approach becoming an increasingly popular and visible component of oncology care. CIM combines complementary practices and conventional medicine in a coordinated way [1]. Complementary and alternative medicine, which is commonly known by the acronym “CAM,” falls within the spectrum of CIM. CAM includes a wide range of products (herbs, vitamins, minerals, and probiotics) and medical practices that denotes both the combined use of complementary or non-mainstream practices with conventional medicine and the use of certain practices instead of conventional medicine [2]: practices and substances are defined as “alternative” when they are used in place of conventional medicine and “complementary” when they are used together with conventional medicine [1, 3].

Recently, the use of mycotherapy, a type of CAM, has been associated with positive impacts in cancer patients in terms of responses to treatment, reductions in side effects, and improvements in quality of life (QoL).

It is estimated that 38% of the general public in the United States (U.S.) uses CAM [4], and 69% of the population of Australia has used at least 1 form of CAM in the past 12 months [5]. A recent Italian survey reported that 49% of cancer patients used CAM during the course of their diseases [3]. One of the most promising types of CAM in cancer patients is mycotherapy, which is derived from traditional Chinese medicine (TCM).

In the year 2000, there were approximately 56 million deaths worldwide from all causes. Cancers were responsible for 12% of these deaths. Roughly 5.3 million men and 4.7 million women developed cancer and 6.2 million people died from the disease. According to the World Cancer Report by the World Health Organization, cancer rates could increase by 50% to 15 million new cases by 2020 [6]. Breast cancer (BC) is one of the most common primary malignancies worldwide, with an estimated 249,260 new cases in the U.S. during 2016 and 40,890 attributable deaths the same year. It is the second leading cause of cancer-related mortality in the U.S. and it has been estimated that 1 in 8 women will develop BC at some point during their lifetimes [7].

Conventional medicine approaches, including chemotherapy (CT), hormone-targeted therapies, immunotherapies, and radiotherapy (RT), are routinely used to treat BC patients: their uses depend on clinicopathological factors, disease stage, and biological characteristics of the tumors. A variety of adverse events are associated with these treatments, such as myelosuppression, gastrointestinal (GI) discomfort and disorders, alopecia, fatigue, infection, cardiotoxicity, respiratory toxicity, and neurotoxicity. These side effects influence patients’ long-term compliance to medications and lower quality of life (QoL) [8]. Due in part to these effects, as well as other reasons, cancer patients are likely to request CAM—in both early and late stages of the disease course: the unfavorable outcomes in a substantial percentage of cases leads patients to ‘leave no stone unturned’ and seek out every option for care, and the heavy toxicities often associated with traditional therapies lead patients to investigate alternatives to the prescribed therapies or, more simply, to seek out substances presumed to reduce the side effects of conventional therapies [3].

One of the most promising integrative approaches in cancer therapy is mycotherapy. This approach appears to have several benefits: (i) improvement in the overall response rate during oncological treatment, (ii) enhanced immunity due to the stimulation of T-lymphocyte proliferation, and (iii) reduction of some adverse events (e.g., nausea and insomnia) due to CT [9]. Medicinal mushrooms have been used for hundreds of years, mainly in Asian countries, for the treatment of infections. More recently, they have also been used in the treatment of pulmonary disease and cancer. They have been approved as adjuncts to standard cancer treatments in Japan and China for more than 30 years and have an extensive clinical history of safe use as single agents or combined with CT.

The anticancer activities of mushrooms have been linked primarily to the modulation of the immune system by branched polysaccharides (glucans), sesquiterpenes, glycoproteins, or peptide/protein-bound polysaccharides [10]. Moreover, mushrooms contain minerals, vitamins (e.g., thiamin, riboflavin, ascorbic acid, and vitamin D), amino acids, and other organic compounds that contribute to the overall health benefits [10]. Some of these natural mushroom compounds have demonstrated specific activity against aberrantly activated signaling pathways in cancer cells and have modulated specific molecular targets of cell functions, including cell proliferation, cell survival, and angiogenesis [10]. These key characteristics of mycotherapy are primarily associated with 2 mushrooms: *Ganoderma lucidum* and *Grifola frondosa* [9, 11–13]. Unfortunately, the small number of studies with high methodological quality limit the application of mycotherapy data.

The purpose of this review was to critically evaluate the described effects of the edible *G. frondosa* (Maitake) and *G. lucidum* (Reishi) mushrooms in patients with BC. Ideally, this review will inspire new scientists to pave the way for new mycotherapy studies in humans, which will begin to bridge the gap between the long history of Eastern medicine and the newer, conventional Western medicine [4–6]. Two authors independently performed a systematic literature search using several electronic databases: PubMed, EMBASE, AMED, Scopus, the Cochrane Library, the National Institutes of Health, and the National Center for Complementary and Alternative Medicine (//:nccam.nih.gov/clinicaltrials/alltrials.htm; accessed June 30, 2017). The search was limited to studies about *G. lucidum* and *G. frondosa* that were published in the English language from January 1971 to April 2017.

## *G. LUCIDUM* AND *G. FRONDOSA*

*G. lucidum* (Curtis) P. Karst. and *G. frondosa* (Dicks.) Gray are considered among the most important medicinal mushrooms in TCM and Japanese medicine. Several studies have examined the biological effects of mushrooms, principally by examining the stimulation of innate immune cells, such as monocytes, natural killer (NK) cells, and dendritic cells (DCs). The activity is generally considered to be caused by the presence of high molecular weight (HMW) polysaccharides in the mushrooms, although other constituents may also be involved [14].

### *G. lucidum* (Reishi)

*G. lucidum* is a mushroom with many interesting bioactive compounds, but some taxonomic discrepancies prevent a clear definition of these compounds: many studies have been conducted to distinguish the European *G. lucidum* s.str. from the Asiatic (Chinese) mushroom. In China and Korea, *G. lucidum* is known as Ling-zhi, which means “spiritual power grass,” while among the Japanese it is called Reishi or mannentake (“10,000 years fungus”). Many authors accept that the Asiatic species is called Ling-zhi fungus and refer to the species as *G. lingzhi* Sheng H. Wu, Y. Cao & Y.C. Dai [15]. *G. lingzhi* seems to have more triterpenic acids than *G. lucidum* s.str. [16–18]. The morphological characteristics of the species also differ [19–21]. *G. lucidum* s.str. basidioma is an annual, laccate, orange-red to dark reddish brown, generally laterally stipitate fungus; the pileus is fan shaped with the upper surface covered by a varnished crust at maturity. *G. lucidum* can be found worldwide in temperate and subtropical areas: it is common in Europe, the Americas (Argentina, Canada, and the U.S.), Africa (Kenya, Tanzania, and Ghana), and Asia (China, Japan, Korea, India, and other southeast Asian countries). It grows at the base of numerous hardwood plants, but rarely on coniferous ones, and mainly on dead wood. Because of its hard texture, it is not an edible mushroom but it is consumed in many ways, the simplest being in the form of teas or herbal teas. Nevertheless, thanks to its varied medicinal properties, it is extensively cultivated in many Asian countries, including China, Korea, and Malaysia. *G. lucidum*, which is a genus of polypore mushrooms, is a leader in terms of worldwide production of medicinal mushrooms.

### *G. frondosa* (Maitake)

*G. frondosa* is another polypore that has a long history of medicinal use. It is commonly known by a variety of appellatives among English speakers, such as “hen of the woods,” “ram's head,” and “sheep’s head”; in Japan, it is known as “Maitake,” a name that means “dancing mushroom.” The origin of the latter nickname is linked to the morphology of the species. The basidioma is large (up to 50 cm wide) and it grows every year in dense clusters at the base of trees. It has a compound structure and appearance, formed by a thick, whitish stipe and a common base from which the whole basidioma extend radially, branching repeatedly and supporting at its extremities individual petaloid basidiomata. The basidioma is constituted by a large number of imbricate, fan-shaped, confluent pilei [22]. The basidioma has a pleasant smell, and, in *1821*, Gray defined this species as edible. It is appreciated for its culinary taste, mainly in Japan, where it is cultivated exclusively for this purpose. Only young basidiomata are edible, since, like all polypores, the fungus becomes tougher as it ages.

*G. frondosa* develops its basidioma around the base of living hardwoods, rarely on conifers. Fruiting can continue on dead trees or stumps. It spreads through submerged, rotting roots, by underground mycelium. It prefers oaks, though it also grows on other deciduous hardwoods such as beech, chestnut, elm, and maple [22]. It grows in northern temperate forests in North America (Canada and the northeastern U.S.), China, and Japan; it is uncommon in Europe.

## BIOLOGICALLY ACTIVE COMPOUNDS

Medicinal mushrooms possess several biologically active compounds (Table 1): HMW compounds such as β-glucans (polysaccharides), glycoproteins, and low molecular weight (LMW) molecules such as quinones, sesquiterpenes, triacylglycerols, isoflavones, catechols, and steroids. Historically, each of these groups of mushroom metabolites was linked to a specific antitumor activity. For example, HMW compounds were believed to exert their antitumor activities by activating the immune response of the host organism, essentially by acting as immunomodulators with effects on both innate and adaptive immunity. LMW molecules were believed to act directly on tumor cells, regulating signal transduction pathways linked to cancer development, progression, and survival. However, increasing evidence shows that some polysaccharides (HMW) also exert direct actions on tumor cells, as observed for Maitake D-fraction (MD-fraction), an extract from the fruit bodies of Maitake that is very rich in proteoglycan β-glucans.

**Table 1:** primarly composition in bioactives substances of *G. Lucidum* and *G. Frondosa*

Substances	Ganoderma	Grifola	Biological Activities
**Water**	6.9%	17,4%	
**Protein**	26.4%	20,3%	Higher nutritional functions
**Fat**	4.5% Stearic acid, Palmitic Acid, Oleic Acid, lignoceric acid, n-nonadecanoic acid, Behenic acid, Tetracosanol, Hentriacontane and Choline triacylglycerols,	3,5 Oleic acid, Stearic acid, Palmitic Acid, lignoceric acid, n-nonadecanoic acid, Behenic acid, Tetracosanol triacylglycerols,	Oleic Acid, an inhibitor of histamine release.
**Food Fiber**	0.1%	0,1%	The dietary fiber of fungus could lower cholesterol level, prevent atherosclerosis, constipation, diabetes.
**Carbohydrate**	43.1% principally Beta-D- Glucan (25%)	46% principally Beta-D- Glucan (20%) which 20% is Maitake D-Fraction	Immunologic, antiinflammatory and anticancer effects involving several pathways via *Dectin-1* receptor (see above).
**Vitamins (mg/100g of product)**	Vit B1 3.5 mg, Vit B2 17 mg, Vit B6 0.7 mg, Choline 1150 mg, Niacin 62 mg, Inositol 307 mg.	Vit B1 3.8 mg, Vit B2 11 mg, Choline 850 mg, Niacin 73.5 mg, Inositol 347 mg, betaine 5.4 mg	
**Terpenes**	Ganoderic acids (Triterpenes)	Various sesquiterpenes	Anti-androgenetic, G1 cell cycle blocking, inhibition of cells adhesion and migration.
**Inorganic (mg/100g of product)**	Calcium 832 mg, Phosphorus 4150 mg, Iron 83 mg, Magnesium 1030 mg, Natrium 375 mg, Potassium 3590 mg,	Calcium 820 mg, Phosphorus 4550 mg, Iron 86 mg, Magnesium 930 mg, Natrium 355 mg, Potassium 3390 mg.	Cyclo octasulfur, a strong inhibitor of histamine release. Exceptional source of Potassium
**Others**	Higher level of the RNA	Moderate level of RNA	RNA inducing interferon production in human cells which disrupts viral invasions
	Isoflavons	High level of isoflavon	Mimic the Estrogen hormones in humans.
**Substances**	*G. Lucidum*	*G.Frondosa*	Biological Activities
**Water**	6.9%	17,4%	
**Protein**	26.4%	20,3%	Higher nutritional functions due aminoacid
**Fat**	4.5% Stearic acid, Palmitic Acid, Oleic Acid, lignoceric acid, n-nonadecanoic acid, Behenic acid, Tetracosanol, Hentriacontane and Choline Triacylglycerols	3.5% Oleic acid, Stearic acid, Palmitic Acid, lignoceric acid, n-nonadecanoic acid, Behenic acid, Tetracosanol triacylglycerols	Oleic Acid, an inhibitor of histamine release.
**Food Fiber**	0.1%	0,1%	The dietary fiber of fungus could lower cholesterol level, prevent atherosclerosis, constipation, diabetes.
**Carbohydrate**	43.1% principally Beta-D- Glucan (25%)	46% principally Beta-D- Glucan (20%) which 20% is Maitake D-Fraction	Immunologic, antiinflammatory and anticancer effects involving several pathways via *Dectin-1* receptor (see above).
**Vitamins (mg/100g of product)**	Vit B1 3.5 mg, Vit B2 17 mg, Vit B6 0.7-mg, Choline 1.150 mg, Niacin 62 mg, Inositol 307 mg.	Vit B1 3.8 mg, Vit B2 11 mg, Choline 850 mg, Niacin 73 mg, Inositol 347 mg, betaine 5.4 mg	General biochemical activities
**Triterpenes**	Ganoderic acids	sesquiterpenes	Anti-androgenetic, G1 cell cycle blocking, inhibition of cells adhesion and migration
**Inorganic (mg/100g of product)**	Calcium 832 mg, Phosphorus 4150 mg, Iron 83 mg, Magnesium 1030 mg, Natrium 375 mg, Potassium 3590 mg	Calcium 820 mg, Phosphorus 4.550 mg, Iron 86 mg, Magnesium 930 mg, Natrium 355 mg, Potassium 3390 mg.	Cyclo octasulfur, a strong inhibitor of histamine release
**Others**	Higher level of the RNA	Moderate level of RNA	RNA inducing interferon production in human cells which disrupts viral invasions
	Isoflavons	High level of isoflavon	Mimic the Estrogen hormones in humans.

Source: //:www.mdidea.net (accessed June 30, 2017).

**Figure 1:**
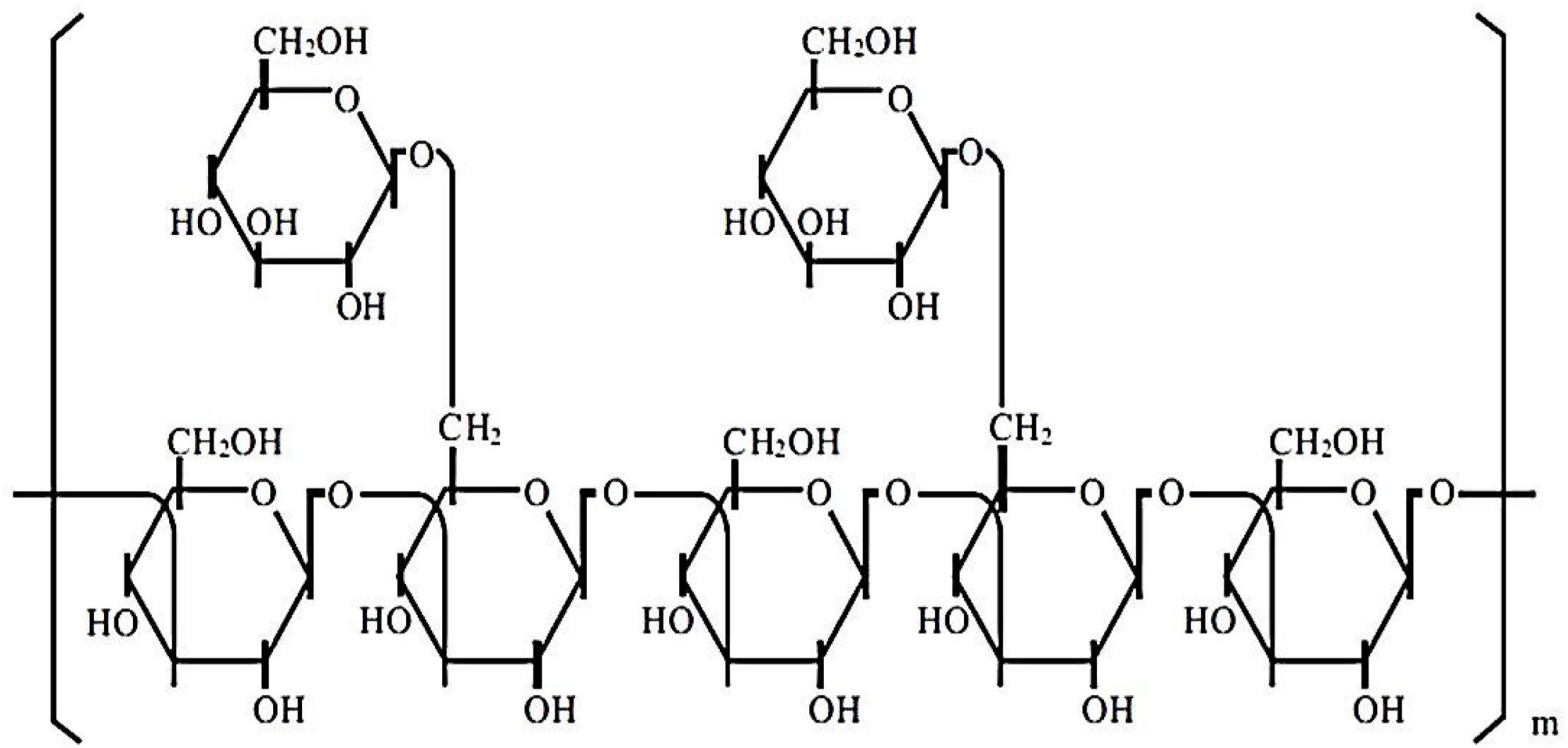
The polymeric structure of β-glucans These molecules are constituted by heterogeneous groups of glucose polymers, consisting of a backbone of β-(1, 3)–linked β-D-glucopyranosyl units with two β-(1, 6) linked side chains every five β-(1, 3)–linked backbone residues.

β-glucans from *G. frondosa* and *G. lucidum* constitute a heterogeneous group of glucose polymers; they consist of a backbone of β-1,3-linked β-D-glucopyranosyl units with β-1,6-linked side chains of varying distributions and lengths (Figure 1). β-1,3-glucans are major structural components of fungal cell walls (Figure 1). These molecular structures have been well identified by crystallographic assay [23]. However, their precise molecular mechanisms of action are still unclear. In particular, the immunomodulatory effects of β-glucans depend on the differences in the degree of branching, polymer length, and tertiary structures among β-glucans [24].

Ganoderic acid (GA) is a major triterpene isolated from G. lucidum in *several isoforms (T, A, Me, H, DM, and X) and its antitumor activity is well-recognized*. In addition, various sesquiterpenes also found with the same antitumor activity. GA effectively inhibits the proliferation of MCF-7 human BC cells via G1 cell cycle arrest. In addition, it significantly decreases the protein levels of cyclin-dependent kinase (CDK) 2, CDK6, cyclin D1, p-Rb, and c-Myc in MCF-7 cells. GA also induces DNA fragmentation and cleavage of PARP, which are characteristic of apoptosis and decrease the mitochondrial membrane potential in MCF-7 cells [25]. GA has anti-androgenic activity, which leads to its therapeutic benefits in prostate cancer, and regulates osteoclastogenesis by suppressing the expression of c-Fos and nuclear factor (NF) of activated T cells c1 [26]. Additionally, GA inhibits proliferation and cell aggregation of HCT-116 cells (a human colon carcinoma cell line) by inhibiting the adhesion of HCT-116 cells to the extracellular matrix in a dose-dependent manner. GA inhibits the nuclear translocation of NF-κB, which leads to downregulated expression of matrix metalloproteinase (MMP)-9, inducible nitric oxide synthase (iNOS), and urokinase-type plasminogen activator [27].

## IMMUNOMODULATING PROPERTIES

**Figure 2:**
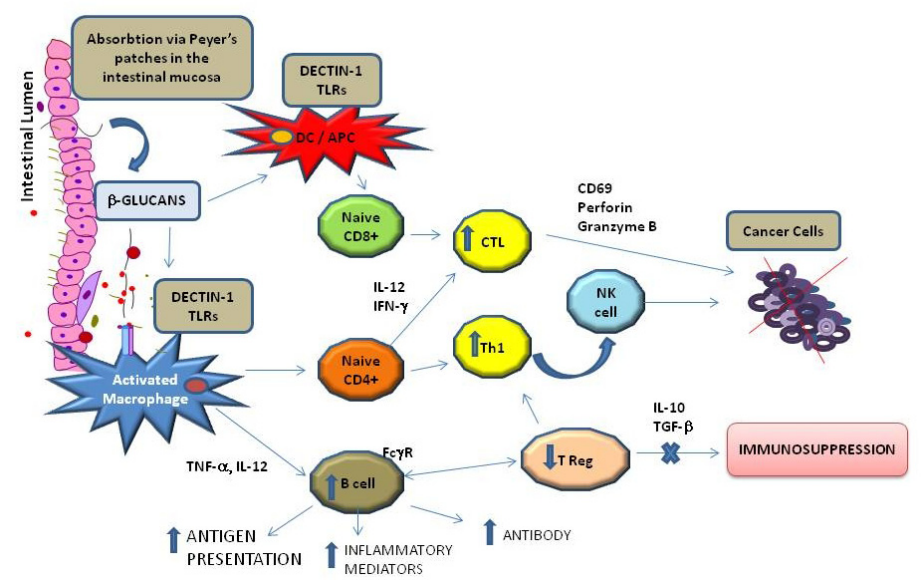
Schematic immunomodulating effect of b-glucans after absorption via intestinal mucosa b-glucans can directly activate macrophages and Dendritic Cells (DCs) in the Peyer’s patches and can induce both, helper T cells (Th) and tumor-specific cytotoxic T. In addition, Fc gamma Receptor (FcgR) provides a critical link between specific humoral responses and cytotoxic T cells Lymphocyte (CTCL).

The functioning of the immune system is critically engaged in the progression of tumors, and immunotherapy is the foremost strategy for cancer treatment. Cancer therapy highlights the role of B and T lymphocytes, DCs, NK cells, and mononuclear phagocyte cells. Mushrooms exhibit interesting immune-regulating properties that may be useful in cancer management. Successful immunotherapy requires both the increase in tumor-specific immunity and the reversal of tumor-associated immune suppression (Figure 2).

Ingestion of oral β-glucans in medicinal mushrooms has been found to activate various immune system components, including macrophages, NK cells, DCs, and T helper lymphocytes, which affects tumor cell viability and potentiates the release of various mediators including lymphokines and interleukins (ILs) [28]. Mammalian digestive enzymes do not digest β-glucans; therefore, it is unclear how soluble β-glucans can be adsorbed in the GI tract. Rice et al [29] demonstrated that 3 fluorescently labeled, soluble β-glucans, which varied in molecular weights and structures, were rapidly absorbed from the GI tract into systemic circulation and detected in plasma after oral administration in rats. Sandvik et al [30] showed that a specific and minute fraction of the orally administered soluble β-glucan translocated to circulation. What is known is that β-glucans are not biomolecules expressed on mammalian cells and, therefore, are recognized as pathogens, similar to viruses, fungi, mushrooms, yeast, algae, lichens, plants, and some bacteria.

Masuda [31] described that the first effect of orally administered MD-fraction, a highly purified soluble β-glucan, is observed in the gut-associated lymphoid tissue (GALT). Orally administered MD-fraction is taken up by antigen presenting cells (APCs) such as DCs and macrophages present in the intestinal mucosa in Peyer’s patches (PPs). MD-fraction, via a C-type dectin-1 pathway, directly induces DC maturation. In this way, the intestinal mucosa could act as a portal for antigen uptake; β-glucans may then be transported to systemic circulation from the intestinal lumen to the lymphoid tissues. The captured MD-fraction is then transported to the spleen, thereby inducing the systemic immune response as demonstrated in tumor-bearing mice [31].

Maitake α-glucan YM-2A isolated from *G. frondosa* has been characterized as a highly α-1,6-branched α-1,4 glucan. Interestingly, YM-2A is more resistant to digestive enzymes than are amylopectin and rabbit liver glycogen: orally administered YM-2A can activate macrophages and DCs in PPs in GALTs. The immunomodulatory effect of YM-2A makes it a promising candidate as an oral therapeutic agent in the translational and clinical research of antitumor immunotherapy [12].

Orally administrated β-glucan is recognized by APCs through multiple interactions with pattern recognition receptors (PRRs), such as the C-type lectin receptor dectin-1 and the complement receptor type 3 [32–36]. Interestingly, the agonists for PRRs, such as the dectin-1 receptors and the toll-like receptors (TLRs), are potent adjuvant treatments for infectious diseases and have been used as immunotherapeutic agents in cancer patients.

Several studies have demonstrated that DCs are functionally defective in tumor-bearing hosts. MD-fraction can directly activate macrophages and DCs and can induce both helper T cells (Th) and tumor-specific cytotoxic T cells to inhibit tumor cell growth [37]. The novel polysaccharide Maitake Z-fraction (MZF), a heteropolysaccharide isolated by Masuda et al, was found to induce DC maturation and antigen-specific Th1 response by enhancing DC-produced IL-12 in murine colon cancer [38]. The authors suggested that MZF could be a potential effective adjuvant therapy to enhance immunotherapy using DC-based vaccination [38].

A proteoglycan bioactive fraction isolated from the fruiting body of *G. lucidum* (GLIS) has been found to strengthen the building of the immune system in mouse spleen lymphocytes, resulting in a 3- to 4-fold increase in the percentage of B cells [39]. The authors suggested that GLIS is a new B cell-stimulating factor [39].

In a non-randomized human clinical trial, 36 patients with advanced (stage II-IV) breast, liver, or lung cancer were administered a combination of oral MD-fraction and Maitake tablets. The treatment demonstrated immune-enhancing properties such as increased numbers of IL-2 and CD4+ cells [40]. Furthermore, it was suggested that β-glucans can cooperate with antitumor monoclonal antibodies that are used for cancer immunotherapy [41]. In fact, when the constant region (Fc) of an immunoglobulin interacts with receptors for the Fc domain of Immunoglobulin G (Fc gamma R [FcγR]) on leukocytes, a variety of biological responses are triggered: phagocytosis, enhanced antigen presentation, release of inflammatory mediators, and antibody-dependent cellular cytotoxicity [28]. Therefore, FcγR provides a critical link between specific humoral responses and cellular immunity, and β-glucans were reported to enhance the expression of FcγR and the activation of complements [41].

The induction of the capacity of native antitumor immune responses is hampered by the immunosuppressive nature of the tumor microenvironment, which is mediated by myeloid-derived suppressor cells and regulatory T cells. The tumor microenvironment also inhibits DC maturation through the secretion of IL-10 and transforming growth factor beta (TGF-β). Cancer cells resist and adapt to inhibit the immune system by releasing immunosuppressive mediators (prostaglandin E2 [PGE2], TGF-β, IL-10, and vascular endothelial growth factor [VEGF]) against immune surveillance. *G. lucidum* and *G. frondosa* polysaccharides counteract this immune inhibition and control tumor formation by suppressing cell proliferation and activation of CD69 expression, perforin, and granzyme B production [42].

In summary, the therapeutic response to orally administered β-glucans *in vivo* is associated with (i) induced systemic tumor-antigen specific T cell response via dectin-1-dependent activation of DCs, (ii) increased tumor-infiltrating, tumor antigen-specific T cells in the tumor, and (iii) decreased tumor-caused immunosuppressive cells such as regulatory T cells and myeloid-derived suppressor cells [31].

## ANTI-INFLAMMATORY PROPERTIES

Mushrooms have been well known for many years in medicine for their beneficial properties based on the modulation of several cytokines and ILs, as demonstrated in both preclinical and clinical studies [9]. Inflammation is a natural process that, when it becomes persistent and of low grade in some tissues, can lead to biochemical reactions in cells activating oncogenes and metabolic changes such as insulin resistance and over-activation of receptor tyrosine kinases (RTKs). In common tumors like breast and prostate cancers, there are small microareas in tissues composed of cancer cells associated with adipocytes, macrophages, and fibroblasts that work together to create a tumor microenvironment that is rich in growth factors, microRNA, and cytokines—all with pro-inflammatory properties that promote survival and chemoresistance [9].

**Figure 3:**
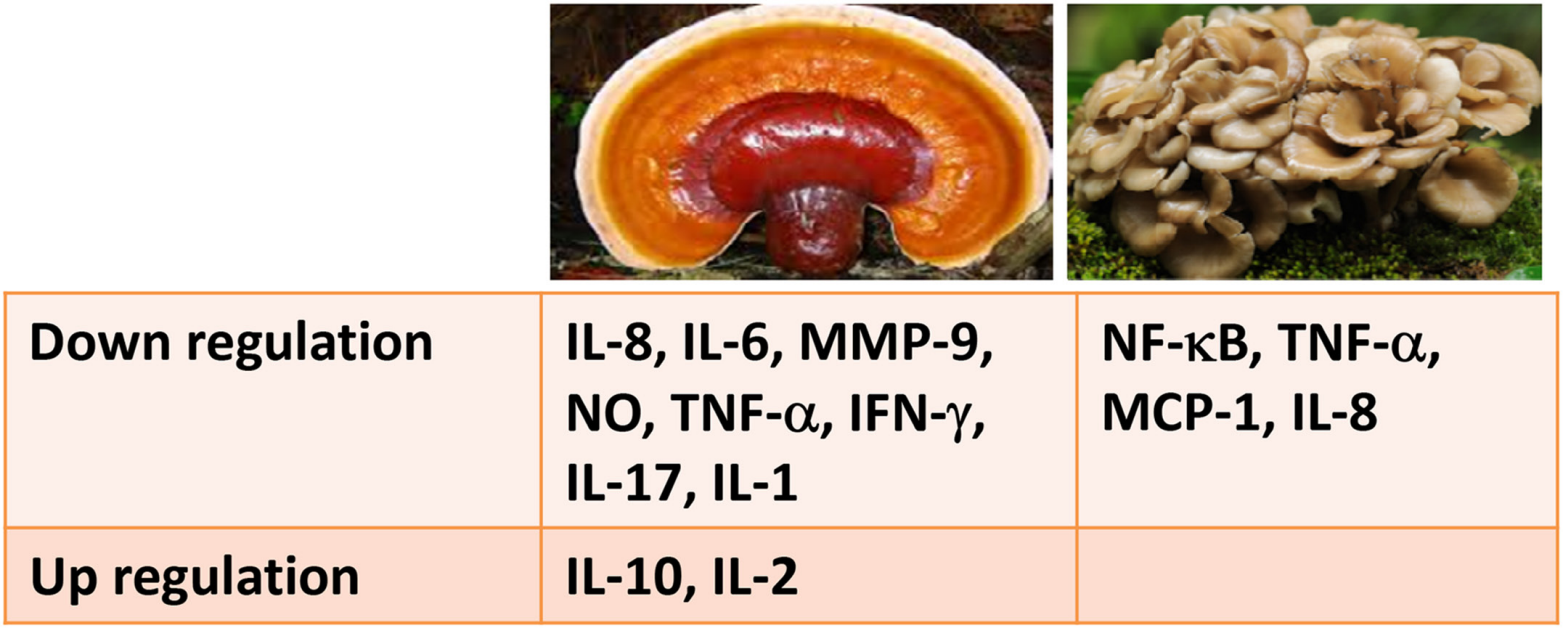
Principal inflammation-involved biomolecules affected by curative mushrooms: *G. lucidum* (left), and *G. frondosa* (right)

β-1,3- and β-1,6-D-glucans extracted from *G. lucidum* are able to decrease inflammation in a concentration- and time-dependent manner. An Indian study demonstrated that *G. lucidum* at a dose of 100 mg/kg body weight showed anti-inflammatory activities comparable to those of diclofenac at a dose of 10 mg/kg body weight, achieving a roughly 50% reduction in inflammation in mouse models. More recently, it was demonstrated that ethanol or DMSO extracts of *G. lucidum* are able to decrease IL-8, IL-6, MMP-2, and MMP-9 secretion by triple-negative human BC cells (i.e., the most aggressive cancer type) exposed to lipopolysaccharides (LPS) by approximately 52%, 50%, 42%, and 50%, respectively, compared to untreated cancer cells [9] (Figure 3). These effects are linked to the downregulation of NF-κB signaling induced by *G. lucidum*. In fact, it was observed that GA C1, one of the most important bioactive compounds present in this mushroom, is able to decrease tumor necrosis factor (TNF)-α, interferon (IFN)-γ, and IL-17A secretion in inflamed colonic cells derived from patients affected by Crohn's disease [43]. From a molecular point of view, many bioactive constituents of *G. lucidum* have shown potent anti-inflammatory effects; in fact, it has been shown to inhibit LPS-stimulated nitric oxide production by macrophages and downregulated mRNA gene expressions of pro-inflammatory cytokines including iNOS, IL-1, and TNF-α in a dose-dependent manner with a marked stimulation of IL-10, a well-known anti-inflammatory cytokine in dopaminergic neurons and microglia [44] (Figure 3).

A recent study demonstrated that *G. frondosa* extract decreased TNF-α levels in rats that received captopril, a common inducer of inflammation and insulin resistance, which suggests a lessening of the inflammatory state in these rats due, most probably, to the β-glucans, flavonoids, ascorbic acid, and α-tocopherol residues present in the extract of the mushroom [45]. Another paper revealed the abilities of *G. frondosa* to enhance INF activity in patients with invasive bladder cancer [46].

The main component of *G. frondosa* extract is a water-soluble glucan/protein (ratio 80:20–99:1) complex [47] that is able to ameliorate colon inflammation by suppressing production of TNF-α and its signaling through NF-κB, which leads to the expression of pro-inflammatory chemokines in HT-29 human colon cancer cells with U937 human monocytic cells (MCP-1 and IL-8).

## DIRECT ANTICANCER ACTIVITY

**Figure 4:**
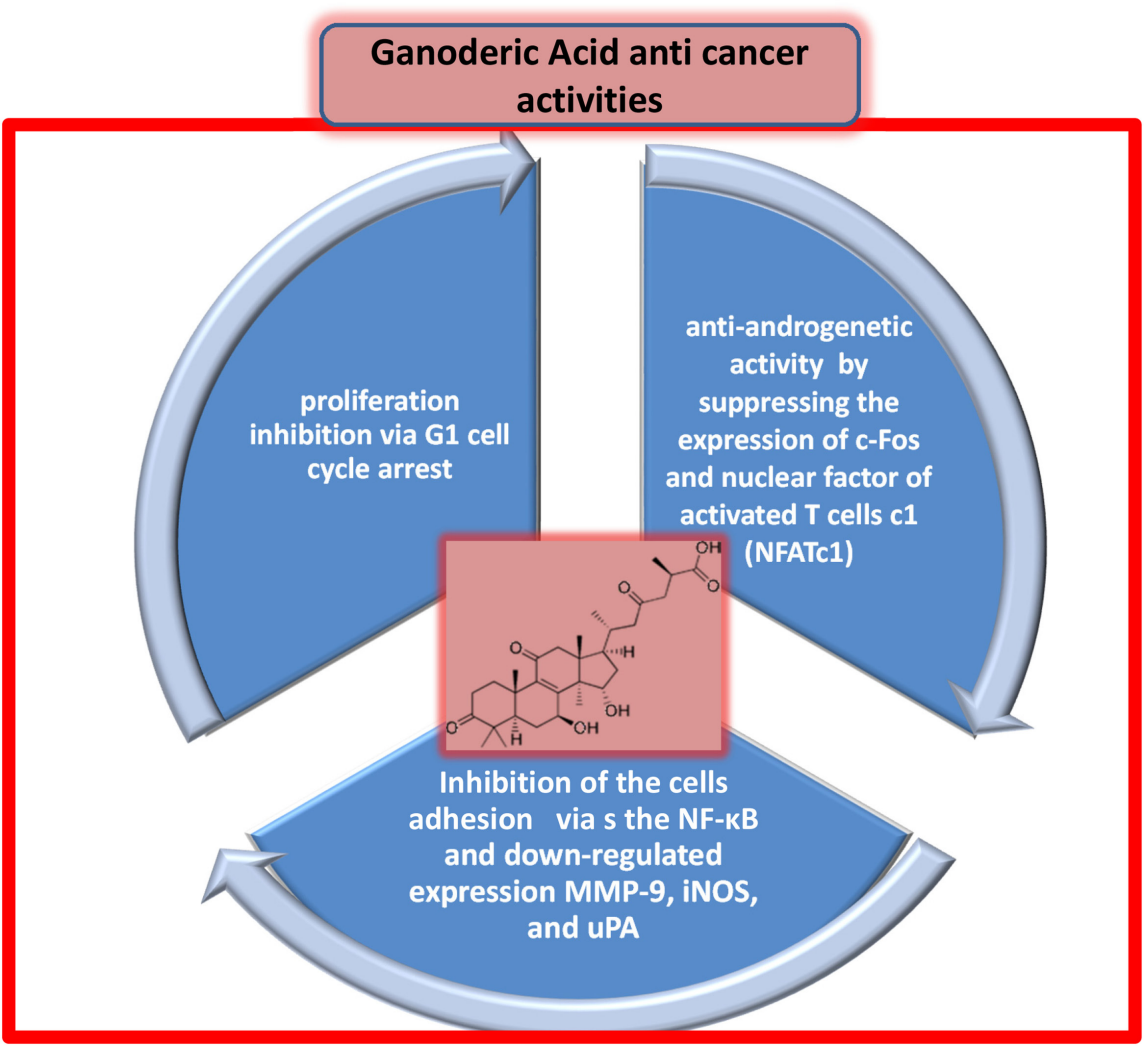
Direct anticancer activities of triterpenes ganoderic acid (chemical structure shown in pink inset)

The anticancer effects of *G. frondosa* and *G. lucidum* have been demonstrated mainly in *in vitro* and *in vivo* experiments (Figure 4), but a very limited number of studies have been conducted in humans. In addition to the intrinsic role of inflammatory processes in cancer’s etiogenesis, progression, and biology, it is essential to consider a holistic point of view in terms of research, as well as clinical practices. From this perspective, mushrooms could be useful during CT or RT, as well as integrated into the diet in the form of supplements, as part of cancer care and management. In fact, several flavonoids, carbohydrates, fatty acids, and glucans derived by mushrooms affect the gene expression and biochemical functions of several oncogenes and tumor suppressors in BC cells [48].

*G. lucidum* modulates the signaling of cancer cells by inhibiting NF-κB nuclear translocation RAS-mitogen-activated protein kinase (MAPK) activation and apoptosis processes. In particular, GA, a triterpene isolated from *G. lucidum*, strongly inhibits the activation of transmembrane surface receptors (RTKs) [49], which modulate intracellular downstream signals responsible for cell adhesion, proliferation, migration, apoptosis, and metabolism in various cancer cells. Specifically, 50 isoforms of GA were studied in relation to specific inhibition of RTKs such as insulin receptor (IR), insulin-like growth factor (IGFR), VEGFR1, VEGFR2, and estrogen receptor in liver cancer cells (Figure 4). Specifically, *G. tsugae* methanol extract was shown to inhibit epidermal growth factor receptor (EGFR) and VEGF, which are important for tumor angiogenesis and growth in human epidermoid carcinoma cells *in vitro* and i*n vivo* [50].

*G. lucidum* has also been evaluated in colorectal cancer *in vitro* and *in vivo*, and in the most common lung cancer biotype. Specifically, *G. lucidum* polysaccharides (GLPS), a water extract of sporoderm broken spores, significantly inhibited the viability of HCT-116 colorectal cancer cells in a dose- and time-dependent manner. GLPS inhibited the functions of RTKs, such as PS-F2, and played a key role in the modulation of MAPKs, JNK, p38, ERK, and NF-κB, which are critical for activation of TNF-α in cancer signaling [51]. Furthermore, GLPS stimulated TNF-α and immunomodulatory activities in lung cancer patients. Oral administration of *G. lucidum* extracts suppressed lung cancer tumor growth in mice and suppressed the activation of PKB (Akt), mechanistic target of rapamycin (mTOR), S6 kinase, and 4E-BP1 in lung cancer cells [52].

The most important preclinical studies of the anticancer effects of Maitake were performed to evaluate MD-fraction activities: it has direct antitumor activity in LM3 murine mammary adenocarcinoma cells, thus preventing oncogenesis and metastasis in human cancer cells [13]. The antitumor effects of MD-fraction have been attributed mainly to its immunostimulatory capabilities, as previously reported, as well as to direct antiproliferative and cytotoxic effects in human cancer cells, including prostate [53], bladder, liver, brain, blood (leukemia), and breast cells [54]. Interestingly, MD-fraction decreases cell viability, increases cell adhesion, and reduces the migration and invasion of human lung cancer cells, generating less aggressive cell behavior [13].

Clinically, Maitake may have the potential to decrease the size of cancers of the lung, liver, and breast. Specifically, in a non-randomized study, a combination of MD-fraction and whole Maitake powder, given alone without any anticancer drugs, exerted anticancer activities in 22- to 57-year-old cancer patients with stage II-IV disease (clinical trial based on different types of cancer). Cancer regression or significant symptom improvement was observed in 58.3% of liver cancer patients, 68.8% of BC patients, and 62.5% of lung cancer patients [55]. On the basis of this information (Table 2), mushrooms can be considered as part of BC patient management to decrease side effects related to CT and increase QoL. Further clinical studies are strongly recommended because, for example, MD-fraction of *G. frondosa* could exert opposite dose-dependent effects under the same immunologic parameters [56]. However, it is interesting that daily oral administration of 5 mg/kg of *G. frondosa* in these patients was associated with the most prominent immunological changes with increasing productions of IL-2, IL-10, TNF-α, and IFN-γ by subsets of T cells.

**Table 2:** Clinical information about how could be recommended the use of *G. lucidum* and *G. frondosa* in hormone dependent and triple negative breast cancer patients

Mushroom	Breast cancer (hormone dependent)	Breast cancer (triple negative)
*G. lucidum*	+++	?
*G. frondosa*	+	?

+++ strongly recommended; + recommended, but need to watch out for possible depressive immune effects in these patients;? no clinical trials found in literature but several mouse models are strongly promising.

## MECHANISM OF ACTION AND PUTATIVE ANTICANCER FUNCTION OF β-GLUCANS IN HUMAN BREAST CANCER CELLS *IN VITRO*

**Figure 5:**
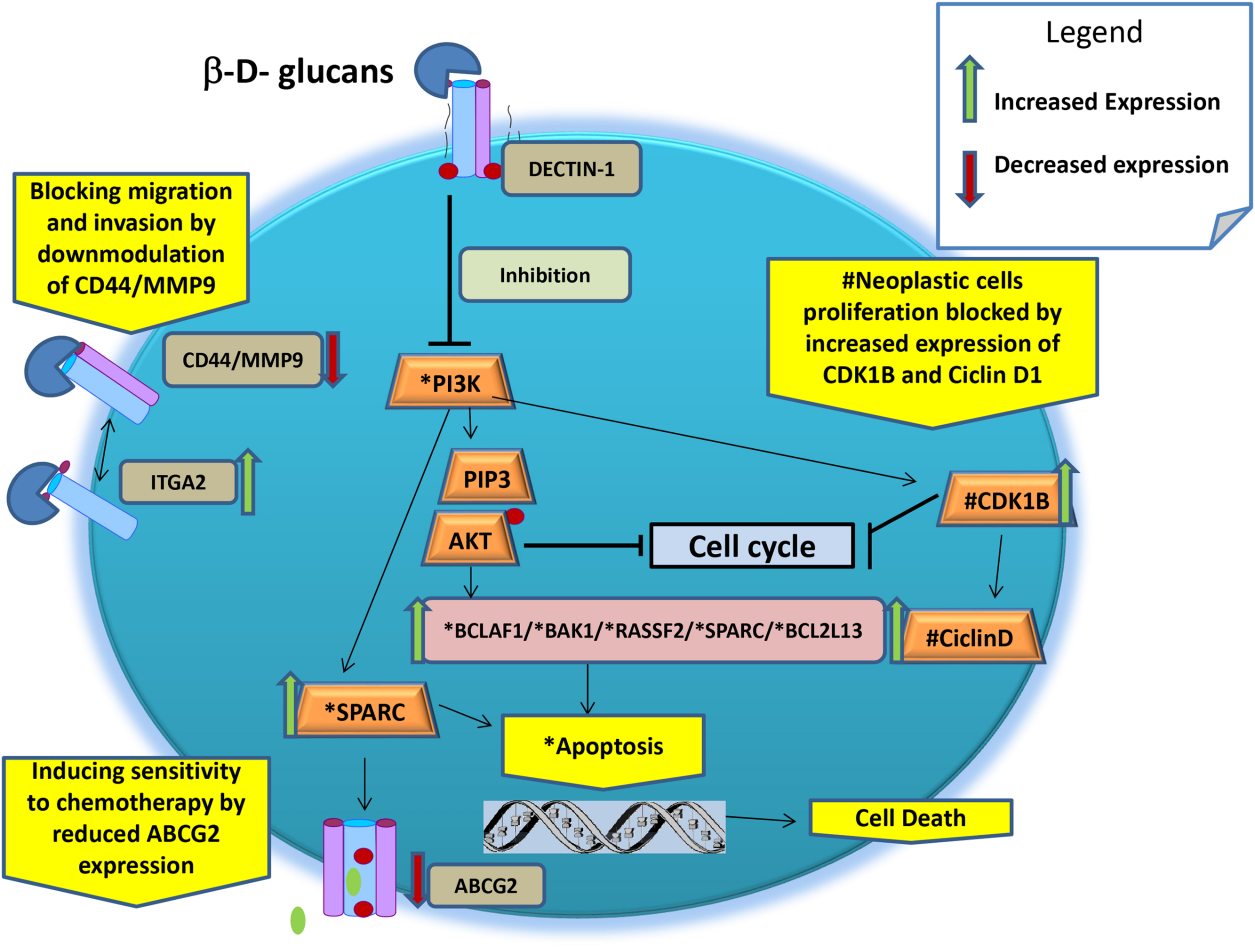
Schematic representation of main putative anticancer cellular mechanisms of β-glucans ^*^ molecules involved directly and indirectly in the apoptosis. ^#^ molecules involved directly and indirectly in the blocking of proliferation.

In a human BC MCF-7 cell model, it was demonstrated that β-glucans can influence the expression of several oncogenes and tumor suppressor genes and, thus, might be able to contrast the BC phenotype developments. Since the cellular mechanisms of actions of β-glucans that influence growth pathways by blocking the *E2F* signals are not yet clarified, we could only describe the effective genotype/phenotype effect mediated by diverse molecular mechanisms, which are postulated below (Table 3 and Figure 5) [13]:

**Table 3:** Synopsis of the Putative Anticancer Function of β-Glucans

Action	^#^Interfering pathway	Annotation	Ref.
**Inducing apoptosis**	Through activation of *BAK1, BCLAF1, RASSF2, FADD, SPARC,* and *BCL2L13* genes and by downregulated PI3K-AKT signalling	The cancer prevention activity primarily depend on the ability of any agents to increases the activation of pro-apoptotic genes which will lead the death of precancerous cells.	[54]
**Blocking neoplastic cells proliferation**	Increases the expression of cyclin-dependent kinase (*CDK*) inhibitor 1B and PTEN/ciclin D1.	Upregulation and downregulation of the cell cycle genes is fundamental for blocking proliferation of neoplastic cells.	[57-59]
**Blocking cell migration and invasion**	Increases expression of α2 integrins (*ITGA2*). Downmodulation of *CD44/MM9* complex	Due to ITGA2 upregulation and *CD44* downmodulation after Maitake treatment, thus blocking tumor cell survival, metastasis, and arteriogenesis in tumoral MCF-7 cells.	[60–62]
**Inducing sensitivity to chemotherapy**	By reducing the expression of ATP-Binding Cassette grouping 2 (ABCG2). Over expression of SPARC	High level of ABCG2 transmembrane protein extrude the high quantity of drugs from the cells, reducing their anticancer activity. SPARC interferes with the interaction between caspase 8 and Bcl2 to resensitize chemo resistant tumors and enhance their regression *in vivo*.	[63]
**Reducing the oxidative stress**	Increasing gene expression of SOD2	Mutant SOD2 drives tumor progression and metastasis by loss mitochondrial anti-oxidative stress functions.	[64, 65]

^#^ referred to gene expression profile studies on cancer cell lines.

a. Inducing apoptosis through activation of BAK1, BCLAF1, RASSF2, FADD, SPARC, and BCL2L13 genes and by downregulating PI3K-AKT signaling. The BAK1 gene product primarily enhances apoptotic cell death after MD-fraction [54]. Activated BAK1 proteins oligomerize at the mitochondrial membrane and cause the release of several mitochondrial factors, such as cytochrome C, which, in combination with pro-caspase 9 and peptidase activating factor 1 interacting protein, starts the apoptotic cascade.

b. Blocking neoplastic cell proliferation through activation of CDK inhibitor 1B (alias p27). It is known that an increase in p27 gene expression is associated with cell growth arrest, cell differentiation, and apoptotic pathways, but its low expression is related to stimulation of cell proliferation and has been demonstrated to have prognostic implications in early stages of mouse mammary pre-neoplasia [57]. Also, β-glucans inhibit tumor cell protein, stimulating the over-expression (4.70-fold) of the retinoblastoma binding protein (RBBP) 4 gene in BC cells [13]. The RBBP4 gene encodes an integral component of transcriptional silencing co-repressor complexes and it is specifically involved in transcriptional repression of E2F-responsive genes. The over-expression of the RBBP4 gene in BC cells blocks the RB-E2F protein complex formation, inhibiting tumor cell growth and proliferation pathways [13]. In addition, IGFR binding protein (IGFRBP)-7 expression is inversely correlated with disease progression in BC cell line MDA-MB-468. These results suggest that the growth of BC could be controlled by the forced expression of the IGFBP-7 protein in human BC cells and xerografted tumors [58]. In addition, it was demonstrated that the upregulation of phosphatase and tensin homolog/p27 complex allowed cell cycle arrest in the G1 phase [59].

c. Blocking cell migration and invasion by increasing the gene expression and activity of α2 integrin (ITGA2) in BC compared to normal breast tissue [60]. Reduced ITGA2 gene expression is highly associated with disease progression and clinical outcomes of BC [61, 62]. On the basis of these findings, we hypothesize that over-expression of ITGA9 and ITGA2 could be involved in the antitumor effects of MD-fraction. In addition, the antitumor activity was tested in MCF-7 BC cells and a 1.47-fold reduction in the expression of the CD44 gene was observed, suggesting that MMP-9 cannot bind the CD44 receptor on the cell surface. Consequently, the CD44/MMP9 complex cannot form, thus blocking tumor cell metastasis and arteriogenesis in the MCF-7 cell line [13].

d. Inducing multidrug sensitivity by reducing the expression of ATP-binding cassette (ABC) group 2 (ABCG2, alias BPRP) [13]. Cells become resistant to chemotherapy through the expression of ABC transporters that, using ATP, extrude different substrates (e.g., cytostatic drugs) out of tumor cells. The ABC transporter BC resistant protein (ABCG2 or BPRP) is a major determinant of the multidrug resistance phenotype. Cancer cells that express low levels of multidrug transporters are more sensitive to CT. In addition, it was found that MD-fraction significantly induces the over-expression (5.45-fold) of osteonectin, also known as secreted protein acidic and rich in cysteine (SPARC) gene, in MCF-7 cells [13]. These findings suggest that increasing the sensitivity to CT might be useful as a complementary therapeutic approach for the subset of triple-negative BC patients who over-express SPARC, as postulated previously [63].

e. Reducing the oxidative stress by increasing the gene expression of superoxide dismutase 2 (SOD2). SOD2 is the key enzyme in mitochondria that protects cells from oxidative DNA damage caused by free radical production. Levels of SOD2 are reduced in many diseases, including cancer [64]. Furthermore, a possible mechanism by which mitochondrial oxidative stress contributes to tumor initiation and progression is an increase in mutation rate that confers putative selection of tumor cell clones with a growth advantage [65].

Still, the exact mechanisms and correlations among these genes and β-glucan is not yet fully understood in humans. However, studies investigating these relationships are crucial for understanding how a nutrigenomic agent and/or CAM can be transformed into a therapeutic agent for cancer [3].

## MICROBIOTA, BREAST CANCER, AND *G. LUCIDUM*

**Figure 6:**
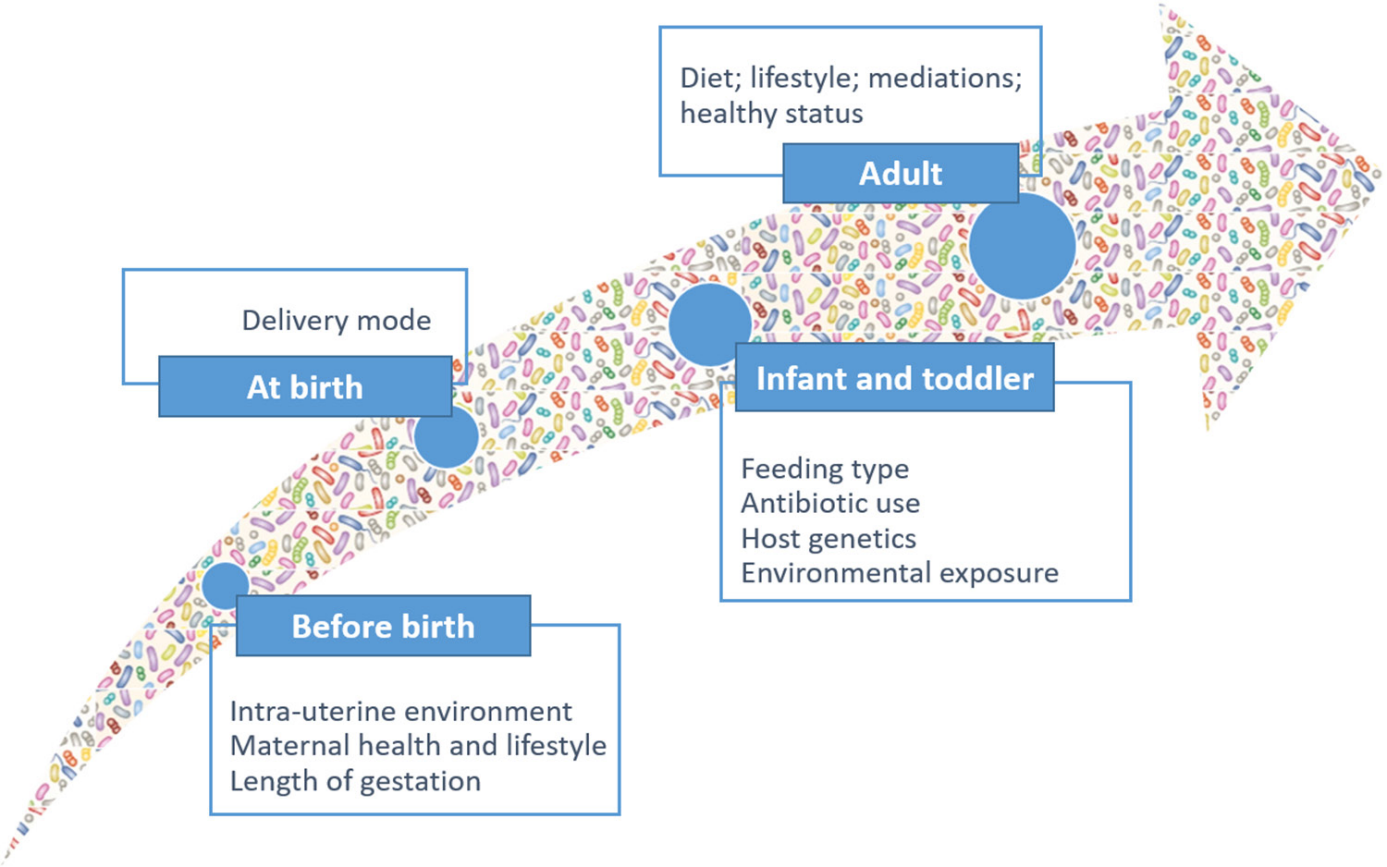
Microbiota dynamics and complexity from birth to adult

Humans are colonized by various commensal microorganisms, which form the human microbiome. The community of microorganisms that exist within the GI ecosystem is termed the “GI microbiota.” To understand the importance of GI microbiota in health and disease, as well as its ability to induce carcinogenesis, it is crucial to appreciate the complexity of its composition, as well as the relationship between microorganisms and host. The adult GI system harbors approximately 100 trillion resident microbes, which represent approximately 10 times the number of cells in the human body [66, 67]. The human GI microbiota is a dynamic and complex ecosystem that contains several different species that cooperate with each other for mutual gains under homeostasis [68]. Microbiota dynamics and complexity increase after birth, reaching the highest points in early adulthood and then remaining quite stable over time (Figure 6).

**Figure 7:**
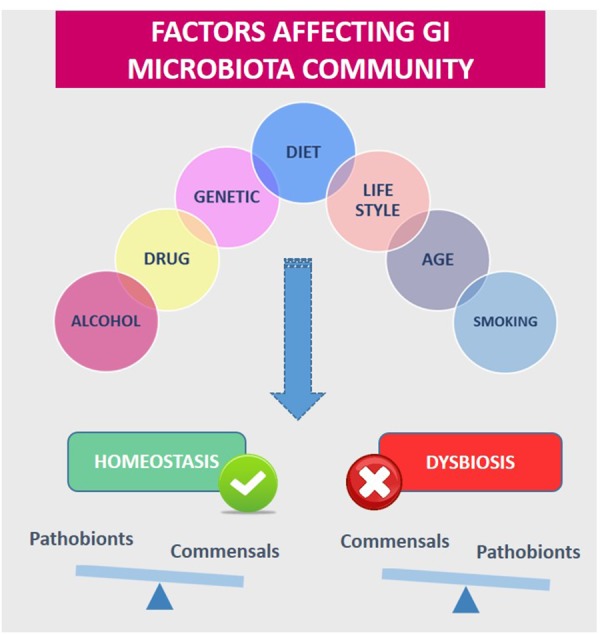
Diet and other environmental factors influence gastrointestinal Microbiota (GI) homeostasis or dysbiosis

However, the microbiota can be affected by diet and other environmental factors such as antibiotic treatment, pathogen exposure, cold stress, or perturbations of diurnal rhythms [69–71] (Figure 7). Changes in the GI microbiota stability and dynamics have been associated with several diseases, including type II diabetes, obesity, fatty liver disease, irritable bowel syndrome, inflammatory bowel diseases, and even certain cancers.

**Figure 8:**
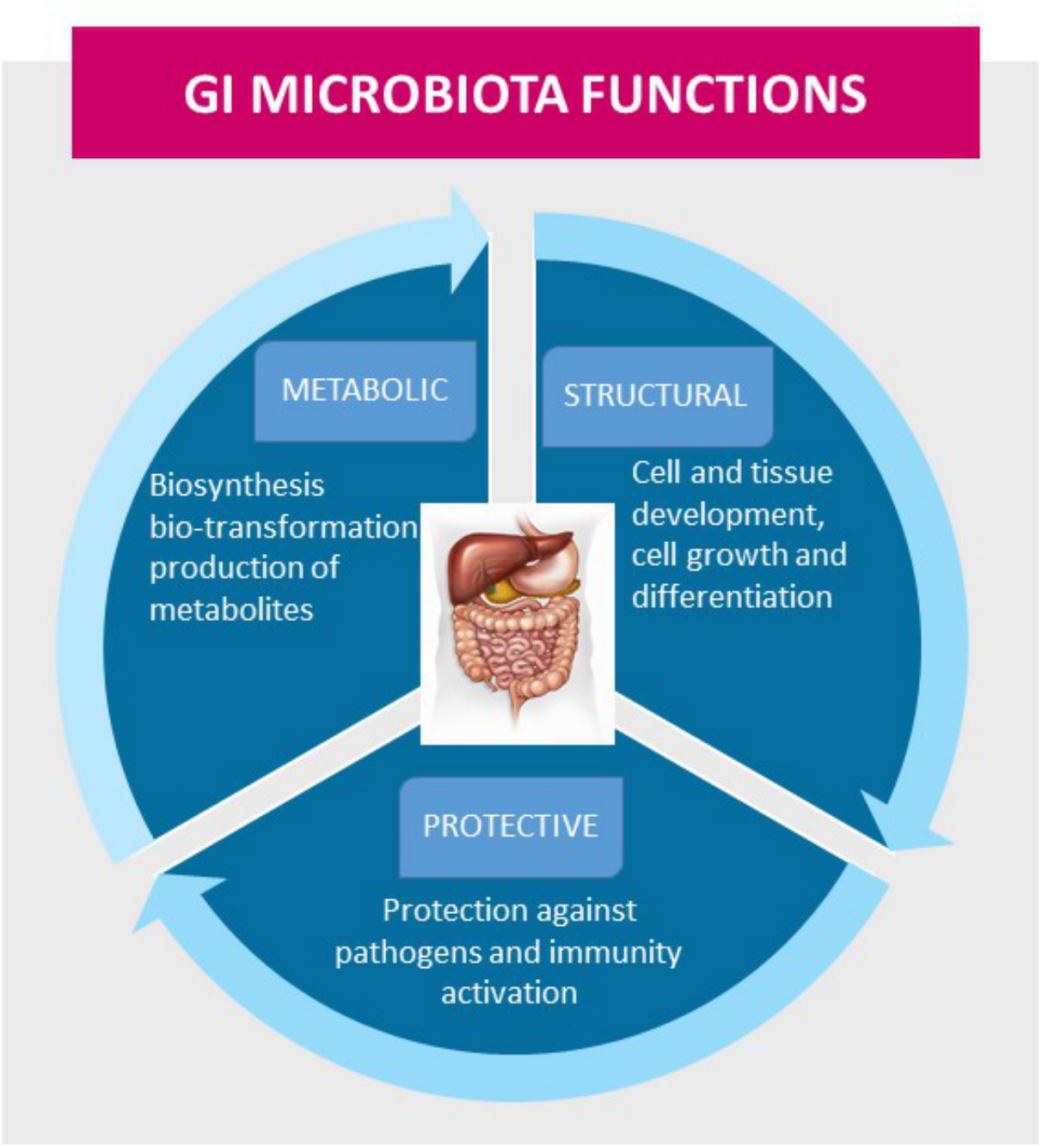
The gastrointestinal (GI) microbiota functions in maintenance of health, including protective, structural, and metabolic roles

The GI microbiota performs essential functions in the maintenance of health, including protective, structural, and metabolic roles (Figure 8). The GI microbiota influences the development of the intestinal barrier and its functions, and it exerts positive stimulatory effects on the intestinal innate and adaptive immune systems, which include the development of the intestinal mucous layer and lymphoid structures, differentiation of immune cells, and production of immune mediators [72]. The link between cancer and microorganisms is well established, and as much as 20% of the global cancer burden has been estimated to be influenced by microorganisms [73]. The mechanisms that contribute to dysbiosis and to alterations in the microbial environment are not yet understood. Host-derived immune and inflammatory responses are important driving forces that shape the composition of the microbial community and, when altered, may contribute to dysbiosis. It is already documented that GI microbiota affects carcinogenesis through the release of carcinogenic molecules, such as genotoxins, and through the production of tumor-promoting metabolites, such as ammonia, amines, phenols, sulfides, and nitrosamines [74–76], which induce responses to DNA damage. It has been suggested that specific low-abundance microorganisms, termed ‘keystone pathogens,’ may further amplify dysbiosis in disease states by exerting dominant effects on bacterial composition [77]. It is clear that bacterial pathogens participate in more than just colorectal carcinogenesis; they affect the development incidence and tumor progression of extra-intestinal cancers, including BC [78, 79]. Recent studies demonstrated that acetate, one of the main SCFA produced by GI microbiota metabolism, can support the growth of several human cancer types, including BC [80, 81]. However, to date, results have not provided a clear understanding of the precise roles that acetate oxidation play in carcinogenesis and cancer progression.

Epidemiological studies have suggested that repeated exposure to broad-spectrum antibiotics induces changes in the GI microbiota, which may affect the metabolism of sex hormones, such as estrogens, thereby influencing the risk of BC [82, 83]. The influence of GI microbiota on BC development, especially in postmenopausal women, is associated with immune regulation, obesity status, and elevated levels of endogenous or circulating estrogens [84–86] through enterohepatic circulation; large differences in these factors exist among individuals [83, 86–89]. Several host factors, including age, ethnicity, antibiotic use, diet, and alcohol consumption, may exert selective pressure on GI microbiota, especially on bacterial species with β-glucoronidase activity (constituents of the “estrobolome”) [90]. In particular, bacterial species belonging to Bacteroidetes and Firmicutes phyla possess distinct β-glucuronidase genes [91–93] that are able to deconjugate the estrogens excreted in the bile, leading to estrogen reabsorption into circulation. This increased total estrogen burden potentially contributes to the risk of development of hormone-driven malignancies such as BC.

Goedert et al. [94] compared GI microbiota profiles and levels of estrogens in urine of postmenopausal BC patients and control patients. They discovered differences between the groups that underscore the relationship between the GI microbiota and hormone-driven carcinogenesis. The active role of GI microbiota in breast carcinogenesis should be investigated further to better elucidate the link between the GI microbiome and estrogen as a driver for BC in order to develop novel therapeutic interventions.

The GI microbiota continue to yield insight into the basic mechanisms of carcinogenesis and several approaches could be used to modulate the carcinogenic potential of the GI microbiota. One strategy could be to push the GI microbiota balance towards richer composition of beneficial bacteria, which can be achieved by alternative approaches such as the use of medicinal mushrooms. There is evidence of potential health benefits of mushroom extracts for their antibacterial, anti-inflammatory, antiviral, antiatherosclerotic, antidiabetic, and anticancer activities [95, 96]. Traditionally, a high number of fungal species have been used as nutraceuticals; among them, one of the most intriguing medicinal mushrooms is the *G. lucidum*, which has been used for centuries to promote health [97]. Mushroom polysaccharides, as well as the products of their partial hydrolysis, are considered prebiotics [98–100], since they may affect the GI tract, binding TLRs expressed in the host [101]. Recently, scientific evidence revealed that *G. lucidum* modulates the composition of GI microbiota in different disorders, such as obesity and hypercholesterolemia, and this ability might contribute to its beneficial effects [102]. Specifically, in an obese mice model, a water extract of *G. lucidum* mycelium was able to maintain the intestinal barrier integrity, reduce metabolic endotoxemia, and reverse high-fat diet-induced gut dysbiosis. These effects were correlated to decreases in both the Firmicutes-to-Bacteroidetes ratio and endotoxin-bearing Proteobacteria levels. In addition, increases in *Roseburia* and Clostridium clusters XIVa and XVIII were also observed [103]. Similar data was reported by Meneses et al. [104], who demonstrated that *G. lucidum* extract modulated GI microbiota composition, with an increase of the *Lactobacillus* genus level in a model of mice fed with a high-cholesterol diet.

Further studies in humans are still needed to better define the mechanisms by which *G. lucidum* can enhance the GI microbiota health. Understanding the physiological mechanisms by which *G. lucidum* exert beneficial effects on GI microbiota would contribute to the success of future clinical trials aimed to investigate its potential for the treatment of GI dysbiosis-related diseases. The assessment of scientific evidence could allow the identification of specific nutraceutical interventions for health improvement by promoting intestinal homeostasis. Within this scenario, it could be interesting to investigate the nutraceutical effects of *G. lucidum* on GI microbiota composition and dynamics of BC patients.

## CONCLUSIONS

Wild mushrooms have been widely used as human food for centuries and have been appreciated for their textures and flavors, as well as for suggested medicinal and tonic characteristics. However, the awareness of mushrooms as a healthy food and as an important source of biologically active substances with medicinal potentials has only recently emerged. Various activities of mushrooms have been studied, including antibacterial, antifungal, antioxidant, antiviral, antitumor, cytostatic, immunosuppressive, antiallergic, antiatherogenic, hypoglycemic, anti-inflammatory, and hepatoprotective activities [95, 105–107]. In this review, we collected evidence of 2 mushrooms (*G. lucidum* and *G. frondosa*) that have potential antitumor properties and, in particular, we focused on these benefits in BC.

It is estimated that half of the 5 million metric tons of mushrooms that are cultivated annually might contain functional or medicinal properties that may be used as a source of biologically and physiologically active substances [108]. Therefore, it is important to understand the isolation, structural characterization, and standardization of mushroom compounds with potential antitumor properties. Macrofungi and, in particular, the so-called medicinal mushrooms, may provide potent compounds with the potential to be used in the prevention and treatment of cancer. However, most of the research reviewed here is relatively recent and has been based on experiments with tumor cell lines or animal models. In fact, findings from many *in vitro* studies and animal models have allowed scientists to begin to clarify the molecular mechanisms involved in anticancer activities. Only a small amount of research has been performed at the level of clinical trials in patients. Therefore, despite the promising published data, more work needs to be completed to define the use of mushrooms, or their isolated compounds, in the prevention and treatment of cancer.

Most of the studies included in this review do not provide clear and unbiased evidence to support the upfront use of G. lucidum in the treatment of cancer patients. There is lack of evidence to support the use of G. lucidum in advanced cancer therapy to improve long-term survival. We do not recommend administration of G. lucidum preparations as a single treatment to patients with metastatic cancer. However, the results of this review suggest that a better response may be expected when a G. lucidum preparation is incorporated as an integrative to conventional CT and/or RT regimens. Therapeutic approaches that incorporate G. lucidum are 1.25 times more likely to yield better tumor response than those do not. Further, G. lucidum preparations can be administered in order to counter the immunosuppressive effects of CT and RT [9], especially in terms of T-lymphocyte depletion. Similar to other natural remedies, G. lucidum is well-tolerated by cancer patients, which leads to better QoL and relatively improved Performance Status (Karnofsky) scores. No severe toxicity has been observed according to current evidence of the use of *G. lucidum*.

Still, current evidence does not support the routine use of G. lucidum in all cancer patients. The decision whether to use a G. lucidum preparation in an anticancer regimen should be made after careful consideration of the cost-benefit potentials and patients’ preferences. Currently, the evidence for using G. lucidum for cancer is sparse and the methodological quality of the trials is poor. As a result, we have only been able to draw a few definite conclusions from the evidence. The lack of standardization in several aspects of the included trials, such as non-uniform preparation and administration of G. lucidum and failure to include key information in the published reports, decrease the reliability and validity of the original trials.

A phase I/II trial of a polysaccharide extract from *G. frondosa* in BC patients demonstrated its activity on some immunologic parameters in the peripheral blood, which has a stimulatory effect on some parameters and a suppressive effect on others. It is interesting to note that no side effects or apparent clinical changes were reported. The most prominent functional changes were increased production of IL-2, IL-10, TNF-α, and IFN-γ by subsets of T cells. The authors concluded that the clinical effects of *G. frondosa* in cancer prevention or treatment remain uncertain.

In conclusion, most of the research reviewed here is relatively recent and has been based on tumor cell lines or animal models. To date, little work has been performed at the level of clinical trials in patients. Therefore, despite the promising published data, more work needs to be completed to clarify the use of mushrooms, or their isolated extracts, in the prevention and treatment of BC.

## MATERIALS AND METHODS

### Literature review

Studies were identified by conducting searches of several electronic databases: the Cochrane Library, Pubmed, EMBASE, AMED, Scopus, the National Institutes of Health, and the National Center for Complementary and Alternative Medicine (nccam.nih.gov/clinicaltrials/alltrials.htm). Eligible studies were published from January 1971 to June 30, 2017.

### Study selection and data extraction

The following search terms were used for the data search: mushroom, breast cancer, immunomodulatory, side effects, Microbiota, *G. lucidum*, and *G. frondosa.* Inclusion criteria also included publication in the English language and publication between 1971 and 2017.

Two researchers (RDF, VQ) reviewed the list of unique articles for studies that fit the inclusion criteria.
